# Long noncoding RNA *Meg3* sponges *miR-708* to inhibit intestinal tumorigenesis via SOCS3-repressed cancer stem cells growth

**DOI:** 10.1038/s41419-021-04470-5

**Published:** 2021-12-21

**Authors:** Shuo Zhang, Wei-Wei Ji, Wei Wei, Li-Xing Zhan, Xuan Huang

**Affiliations:** 1 Department of Gastroenterology, The First Affiliated Hospital of Zhejiang Chinese Medical University, Hangzhou, 310006 China; 2 CAS Key Laboratory of Nutrition, Metabolism and Food Safety, Shanghai Institute For Nutritional Science, Shanghai Institutes for Biological Sciences, Chinese Academy of Sciences, Shanghai, 200031 China

**Keywords:** Cancer stem cells, Stem-cell research

## Abstract

**Background:**

Colorectal cancer (CRC) remains the most common gastrointestinal cancer and a leading cause of cancer deaths worldwide, with most showing pathologies indicating the malignant transformation of early stage intestinal stem cells. The long non-coding RNA *Meg3*, which functions as a tumor suppressor, has been reported to be abnormal in multiple tumorigenesis events; however, the underlying mechanism by which *Meg3* contributes to the malignant proliferation of colonic stem cells remains unclear.

**Methods:**

We analyzed the expression levels of *Meg3*, *miR-708*, and SOCS3 in samples from *Apc* loss-of-function (*Apc*^*min*^) mice and patients with CRC, particularly in colonic crypt cells. *Apc*^*min*^ mice and AMO/DSS-induced mice model (in vivo) and organoid culture system (in vitro) were used to explore the effect of the *Meg3*/*miR-708*/SOCS3 axis on tumorigenesis in the colon. In vitro, we performed RNApull-down, RNA immunoprecipitation, and luciferase reporter assays using DLD1 and RKO cell lines.

**Findings:**

The *Meg3*/*miR-708*/SOCS3 signaling axis plays a critical role in the early stage of CRC development. Our data showed *Meg3* levels negatively correlate with *miR-708* levels both in clinical samples and in the *Apc*^*min*^ mouse model, which indicated that *Meg3* acts as a competitive endogenous RNA (ceRNA) of *miR-708*. Then, *miR-708* served as an oncogene, inducing neoplasia in both *Apc*^*min*^ mice and cultured colonic organoids. Put together, *miR-708* appears to promote malignant proliferation of colonic stem cells by targeting SOCS3/STAT3 signaling.

**Interpretation:**

These data revealed that *Meg3* sponges *miR-708* to inhibit CRC development via SOCS3-mediated repression of the malignant proliferation of colonic stem cells. The *Meg3*/*miR-708*/SOCS3 signaling axis provides potential targets for the diagnosis and treatment of CRC, particularly early stage CRC.

## Introduction

Colorectal cancer (CRC) remains the most common gastrointestinal cancer and a leading cause of cancer deaths worldwide [1–3]. Notably, the incidence and mortality of CRC continues to increase globally in recent decades [1–3]. Clinically, the most critical prognostic factor is the pathological stage of CRC at diagnosis [3]. Though the application and popularization of colonoscopy and the development of effective biomarkers has permitted remarkable progress in CRC diagnosis and therapy, the advanced CRC prognosis and diagnosis of early stage CRC remain major unmet medical needs. Therefore, it is urgent to explore the fundamental processes of CRC to identify more effective diagnostic and therapeutic targets for interventions against advanced colon polyps and early stage CRC.

Intestinal stem cells (ISCs), which are localized at the bottom of intestinal crypts, have been identified as central in CRC, such that malignant transforming events in ISCs contribute to the initiation of most intestinal cancers [4, 5]. Lineage retracing has revealed that almost 75% of adenoma cells originate from Lgr5^+^ ISCs [6]. Consequently, it is necessary to investigate the underlying mechanism of malignant transformation of ISCs in early stage CRC.

Epigenetic changes, including alterations of noncoding RNA (ncRNA), are considered important contributors to tumorigenesis; epigenetic alterations in CRC occur earlier and more frequently than do genetic alterations [7, 8]. MicroRNAs (miRNAs) and long ncRNAs (lncRNAs) are the two most commonly studied ncRNAs in CRC. Notably, an increasing body of literature has identified miRNAs as clinically useful biomarkers for diagnosis and therapy of CRC; and emerging evidence suggests that dysregulated lncRNAs have functional effects on CRC pathogenesis, although the underlying mechanisms of most lncRNAs in CRC remain incompletely understood [7–9]. lncRNAs may serve as competitive endogenous RNAs (ceRNAs), “sponging up” miRNAs to deplete their level and thereby alter the expression of the miRNA’s target genes [10]. Thus, the role of the lncRNA-microRNA axis in CRC has been the subject of recent attention, although the specific contribution of this axis to the malignant transformation of ISCs in CRC is unknown. Efforts devoted to elucidating the underlying mechanisms are essential and warrant further exploration; such research is expected to provide promising biomarkers for the effective diagnosis and therapy of both early stage and advanced CRC.

A maternally expressed gene (*Meg3*) acts as a tumor suppressor in multiple cancers including CRC [11]. *Meg3* levels are decreased in patients with CRC (compared to control patients); higher *Meg3* levels have been shown to positively correlate with better overall survival and disease-free survival in CRC [12, 13]. Accumulating evidence indicates that *Meg3* is involved in cell proliferation, migration, invasion, and chemoresistance in CRC via “sponging” of mRNAs or miRNAs [13–15]. *Meg3* depletion has been shown to strengthen stem-cell-like characteristics in diverse cell types, including lung cancer cells, mesenchymal stem cells, and germline stem cells [16, 17]. However, the effects of *Meg3* in early stage CRC and malignantly transformed ISCs remain unclear.

Herein, we demonstrated a sponging role for *Meg3* in the *miR-708*/SOCS3 axis, affecting the colonic stemness of early stage CRC. Firstly, we surveyed the miR-omes of colonic crypt cells with different *Meg3* expression profiles using *Apc*^*min*^ mice (a strain that harbors a germ-line mutation in Apc, the mouse homolog of the human APC gene, which renders the animals prone to developing CRC); this work revealed that *miR-708* is an important component in *Meg3*-related colonic stemness. Both in a mouse model and in the clinic, higher *miR-708* levels indicated progression to later stages of CRC and poorer overall survival. In addition, *Meg3* level negatively correlated with *miR-708* level in CRC. Functionally, *miR-708* is capable of promoting colonic adenoma development in vivo and accelerated colonic organoid and CRC cell growth in vitro. Mechanistically, *Meg3* sponged *miR-708* to hijack organoid and CRC cell growth through SOCS3-induced growth inhibition. Notably, the sponging correlation among *Meg3*, *miR-708*, and SOCS3 were present in a colonic adenoma mouse model and in clinical CRC tissues. Collectively, the current study revealed the role of the *Meg3*/*miR-708*/SOCS3 axis in determining colonic stemness in early stage CRC, demonstrating that targeting the combination of *Meg3* and *miR-708* might represent a diagnostic and therapeutic strategy in early stage CRC.

## Materials and methods

### Mice and mouse model

All animals were bred, maintained, and used in accordance with the guidelines of the Institutional Animal Care and Use Committee of the Shanghai Institute of Nutrition and Health (SINH) (Shanghai, China). All mice were housed under specific-pathogen-free conditions in laboratories maintained at 23 ± 3 °C with a relative humidity of 35% ± 5% and a 12-h/12-h dark/light cycle. Throughout the study, animals were provided with free access to a standard diet (Shanghai Laboratory Animal Co., Ltd., Shanghai, China) and drinking water, except as noted below.

To generate *miR-708*^−/−^ mice, the Sanger MirKO ES cell line miR-708 was microinjected into C57BL/6 mice at Shanghai Model Organisms (Shanghai, China); resulting male chimeric mice were crossed with C57BL/6 females to generate heterozygous mice, which then were crossed to generate *miR-708*^+/+^ (wild-type, WT) and *miR-708*^−/^^−^ (knockout, KO) mice. WT and KO mice were co-housed until 4 weeks of age.

For the *Apc*^*min*^ mouse model, WT or KO mice (8 weeks old, female) were crossed with *Apc*^*min*^ mice (8-week-old male) to generate *Apc*^*min*^ *miR-708*^+/+^ (wild-type) mice or *Apc*^*min*^ *miR-708*^−/−^ mice. Male mice were used in colonic crypt isolation (from 8-week-old animals) and for the development of early stage CRC.

For the azoxymethane/dextran sulfate sodium (AOM/DSS)-induced colorectal cancer model, the mice were treated as previously described [18]. Briefly, mice (6–8-week-old-male) were injected subcutaneously with AOM (10 mg/kg) on Day-5, and then provided with free access to 3% DSS (160110, MW = 36–50 kD; MP Biomedicals, Santa Ana, CA, USA) in the drinking water for 5 days starting on Day 0 (i.e., starting 5 days after administration of AOM). After the 5th day on 3% DSS, the animals were shifted back to standard (unadulterated) drinking water. DSS treatment was repeated for 3 cycles every 16 days. To increase *Meg3* levels or decrease SOCS3 levels in colon tissues, *Meg3* adenovirus (Adv-Meg3), SOCS3 interference adenovirus (Adv-SOCS3i), or control adenovirus was administered to mice (5-week-old-male) by rectal instillation (per injection/7days).

All animal experiments were approved by the Institutional Animal Care and Use Committee of the Shanghai Institutes for Biological Sciences, Chinese Academy of Sciences (Shanghai, China).

### Human cohort

This study was approved by the Institute Research Ethics Committee of the First Affiliated Hospital of Zhejiang Chinese Medical University. Two validated cohorts of patients with CRC were enrolled in this study. For each cohort, patients must have been pathologically confirmed with CRC. Tumor specimens from these patients were obtained at the time of surgical resection before therapy. Paired samples (tumor and adjacent normal tissue), embedded by formalin-fixed paraffin, were obtained from these patients who underwent operations and matched all clinicopathologic variables.

### Colonic crypt isolation and organoid culture

Following euthanasia of 8- to 10-week-old male AOM/DSS mice, the entire colons were recovered, everted, and rinsed 5 times with ice-cold phosphate-buffered saline (PBS) supplemented with 2% (vol/vol) penicillin and streptomycin. Colonic crypts were isolated by immersing the everted and rinsed colons in chilled (0–4 °C) Cell Recovery Solution buffer (Corning Cat. 354253; Corning, NY, USA) for 30–40 min before passage through 70-μm strainers to filter the resulting cell suspension. The suspended cells then were rinsed 3–4 times with basic cell culture medium (DMEM/F12; Gibco^TM^, Waltham, MA, USA) supplemented with 1% (vol/vol) penicillin and streptomycin (Gibco^TM^), 1% GlutaMAX (Gibco^TM^), and 1% HEPES buffer (4-−1-piperazineethanesulfonic acid; Gibco^TM^). The isolated colonic crypts were resuspended in Matrigel (BD Cat. 354230; BD) and plated in pre-warmed (37 °C) 24-well culture plates, which then were incubated in a cell culture incubator (at 37 °C, in a humidified atmosphere with 5% CO_2_) for 10 min after seeding. Colon organoid growth medium (consisting of a 1:1 mixture of DMEM/F12:L-Wnt 3 A supernatant (see below) supplemented with 20% (vol/vol) fetal bovine serum (FBS; BI), 1% (vol/vol) penicillin and streptomycin, 500 ng/mL R-Spondin 1 (Cat. 3474-RS, R&D), 50 ng/mL epidermal growth factor (EGF; Cat. 50482-M01H; Sino Biological Co., Ltd., China), 100 ng/mL Noggin (Cat. 250-38-20, Peprotech), 10 μm Y-27632 (Cat. 1254; Tocris)) was distributed to the culture plates at 500 μL/well; the day of the addition of this growth medium constituted the nominal Day 0 for organic growth. After culture for the indicated times, organoids were subjected to western blot or quantitative reverse transcription-polymerase chain reaction (qRT-PCR) analysis.

For passaging, organoids were washed with cold PBS. After centrifugation at 200×*g* for 2–3 min, the organoids were resuspended with Matrigel (50 μL/well) and then plated. For *miR-708* agomir, *miR-708* antagomir, or *Meg3* interference studies, the agomir, antagomir, or adenovirus was added to the growth medium at Day One.

The L-Wnt 3A cell line was purchased from Shanghai Fuxiang Biotechnology Co., Ltd. and cultured in growth medium (DMEM supplemented with 10% FBS and 1% (vol/vol) penicillin and streptomycin) to 80–90% cell confluence; the resulting conditioned medium was collected every three days for a total of three times and filtered through 0.22-μm filters. The diameter and the number of organoids were measured by ImageJ.

### Cell lines and cell culture

The colorectal cancer cell lines (DLD1 and RKO) and the IMEC cell line were obtained from our laboratory collection. All of the above cell lines were cultured with DMEM medium (Life Technologies) supplemented with 10% FBS and 1% penicillin/streptomycin and incubated at 37 °C in a humidified atmosphere with 5% CO_2_.

### Cell proliferation assay

DLD1 or RKO cells (4000/well) were plated in 96-well plates in triplicate. The Cell Counting Kit 8 (CCK8) (Abcam) was used to measure cell proliferation. We performed this assay according to the manufacturer’s protocols.

### Western blot

Western blot assays were performed as described previously [19]. Immunoblotting in the present study employed antibodies (purchased from Cell Signaling Technology, Boston, MA, USA) against SOCS3, Stat3, p-Stat3(Tyr705), Hsp90, and the housekeeping protein glyceraldehyde phosphate dehydrogenase (GAPDH); GAPDH and Hsp90 were used as a loading control.

### RNA extraction and qRT-PCR

RNA extraction and qRT-PCR were performed as described previously [19]. The primers used in this study are shown in Table 1.

**Table 1:** qRT-PCR primer sequences.

Gene	Forward primer 5′-3′	Reverse primer 5′-3′
hSOCS3	CCTGCGCCTCAAGACCTTC	GTCACTGCGCTCCAGTAGAA
mSOCS3	TGCGCCTCAAGACCTTCAG	GCTCCAGTAGAATCCGCTCTC
hMeg3	CATCTACACCTCACGAGGG	ATCCTTTGCCATCCTGGTC
mMeg3	TCCTGGATTAGGCCAAAGC	AGCCTATTTGAGAAGCTGGT
MiR-708	CGTCAAGGAGCTTACAATCT	CAGTGCGTGTCGTGGAGT
mGAPDH	AGGTCGGTGTGAACGGATTTG	TGTAGACCATGTAGTTGAGGTCA
hGAPDH	CTGGGCTACACTGAGCACC	AAGTGGTCGTTGAGGGCAATG

### Hematoxylin and eosin (HE) staining, immunohistochemistry (IHC), immunofluorescence (IF), and RNA-fluorescence in situ hybridization (RNA-FISH)

HE staining, IHC, and IF were performed as described previously [19, 20]. IHC employed the following reagents: anti-Ki67 antibody (Novus Cat. NBP1-40684), anti-SOCS3, and anti-p-Stat3 antibodies (Cell Signaling Technology). IF employed the following reagent: anti-p-Stat3 antibody (Cell Signaling Technology).

For the RNA-FISH assay, the colonic tumor tissues from *Apc*^+/^^*min*^ mice were recovered at necropsy, washed with PBS, fixed in 4% PFA buffer (paraformaldehyde diluted in PBS that had been pre-treated with Diethylpyrocarbonate). The fixed tissues then were embedded in paraffin. After sectioning with an Ultracut microtome (Leica, Bannockburn, IL, USA), the embedded tissues were dehydrated and digested with Protein K, pre-hybridized for 1 h, and hybridized with *miR-708* probe overnight at 37 °C. The *miR-708* probe consisted of a 6-carboxyfluorescein (FAM)-labeled oligonucleotide with the sequence 5′-FAM-GGGUCGAUCUAACAUUCGAGGAA-FAM-3′; the oligo was purchased from Guangzhou Ribobio Co., Ltd. (Guangzhou, China). After incubation with 4′,6-diamidino-2-phenylindole(DAPI) staining buffer for 10 min, the sections were mounted and then examined with an Olympus IX81 fluorescent microscope.

### RNA pull-down assay

The RNA pull-down assay was performed as described previously [21]. *miR-708* or *Meg3*, labeled with biotin, was transfected into cells for 24 h, followed by incubation of the cell lysates with M-280 streptavidin magnetic beads (Sigma). Finally, the *Meg3* or *miR-708* level was measured by qRT-PCR.

### Luciferase reporter assay

We performed the luciferase reporter assay as described previously. Briefly, the DNA sequences corresponding to the 3′-UTR of the *SOCS3* or *Meg3* transcript, which contain putative *miR-708* binding sites, were amplified and cloned into the pGL3 vector. Mutation of the sequences at the specific putative binding sites was performed using the Quick Mutation^™^ Site-Directed Mutagenesis Kit (Beyotime Biotechnology, China). For the *Meg3*-related luciferase reporter assay, the *Meg3*-WT or Mut luciferase vector and the *miR-708* antagomir were co-transfected into DLD cells for 24 h; separately, the *Meg3*-WT or Mut luciferase vector was transfected into RKO cells (which exhibit constitutive *miR-708* expression) for 24 h. For the *SOCS3*-related luciferase reporter assay, the *SOCS3* 3′UTR-WT or Mut vector was co-transfected with either the control agomir or the *miR-708* agomir into cells pre-treated with Adv-Meg3 for 24 h; separately, the *SOCS3* 3′UTR-WT or Mut vector was co-transfected with either the control antagomir or *miR-708* antagomir into cells pre-treated with Adv-Meg3i for 24 h. Following transfection, luciferase activity was assessed using a dual-luciferase reporter assay system (Promega, USA); and the relative luciferase activity was normalized to that of Renilla luciferase activity.

### RNA immunoprecipitation (RIP) assay

The RIP assay was performed using the Magna RIP Kit (Millipore) according to the manufacturer’s protocols. Briefly, cells that had been transfected with the *miR-708* antagomir, *miR-708* WT, or *miR-708* Mut for 24 h were subjected to the RIP assay; cell lysates then were incubated with antibodies against IgG or AGO2. *Meg3* levels in the RIP products were detected using qRT-PCR.

### Statistical analysis and bioinformatics analysis

*miR-708* targets and the paired sites of *Meg3* were predicted using the online websites miRbase (http://www.mirbase.org), Targetscan (http://www.targetscan.org), and miRDB (http://www.miRdb.org). CRC datasets were obtained from GEO datasets. Experimental data are presented as the mean±S.D and were analyzed by unpaired, two-tailed Student’s *t* tests; analyses were conducted using Prism 6.01 software (GraphPad; San Diego, CA). *p* Values of less than 0.05 were considered significant.

### Generation and administration of recombinant

Adenoviruses Recombinant adenoviruses expressing Meg3 (Adv-Meg3), SOCS3 (Adv-SOCS3), and Interfering AF6 (Adv-Meg3i), SOCS3 (Adv-SOCS3i) were constructed using the AdMax system (OBiO Technology) according to the manufacturer’s instructions. Purified high-titer stocks of amplified recombinant adenoviruses were diluted in PBS and administered at a dose of 1 × 10^7^ plaque-forming units (PFU)/well in 12-well plates or injected at a dose of 1 × 10^9^ PFU/mouse through coloclysis. Injection of the adenoviruses above or control viruses did not affect food consumption compared with that of control-treated animals.

### Flow cytometry

Organoids were dissociated into a single with Accutase, and large clusters were removed with a 40 Micrometer Cell Strainer (Corning). The cells were washed with cold PBS and then fixed with Fixation/Permeabilization buffer (Biogems) at 4 °C for the night. The Cells were incubated with the LGR5 antibody(Abcam) at 1/25 dilution at 37 °C for 60 m. Then Cells were incubated with the second antibody at room temperature for 30 m. The cells were suspended with flow cytometry staining buffer, then assayed using a Flow cytometer.

### Soft agar assay

Totally, 1 × 10^3^ cells were inoculated into 0.35% agar containing 1× medium and seeded in each well of a 6-well plate containing 0.6% agar in 1× medium. Cells were grown at 37 °C for 21 days. Cells were fixed with 4% paraformaldehyde and stained using 0.1% crystal violet. The cells were imaged and quantified with ImageJ.

## Results

### Meg3 levels negatively correlate with miR-708 levels in colonic crypts of Apc^*min*^ mice

**Fig. 1:**
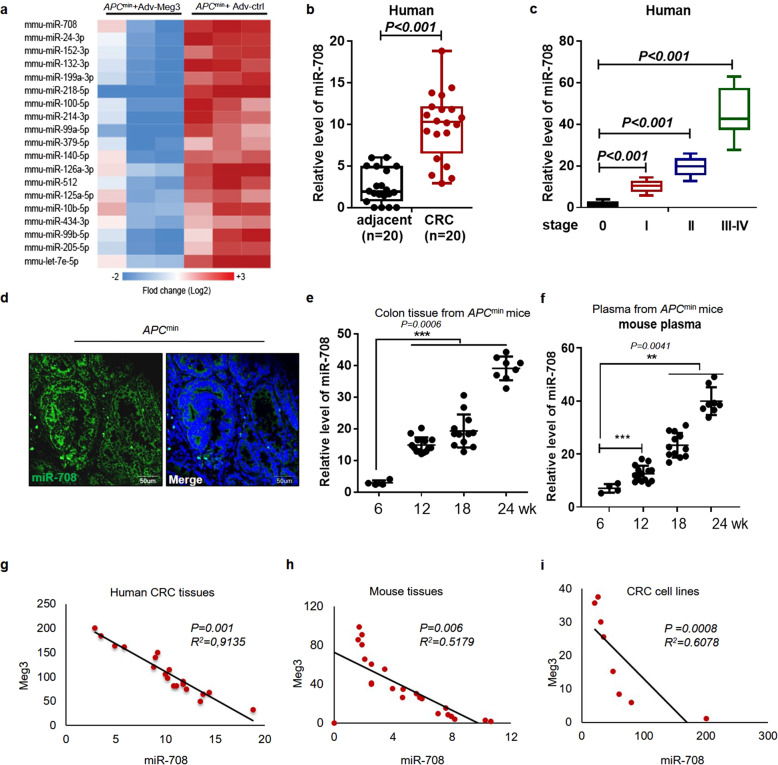
The level of *Meg3* negatively correlates with that of *miR-708* in colonic tumorigenesis. **a** A heat map showing miRs with significant accumulation (fold change > 2, *p* < 0.05) in colonic crypts from *Apc*^*min*^ mice (12 weeks old) administered (rectal instillation) with the *Meg3*-overexpressing adenovirus (Adv-Meg3) or control adenovirus (Adv-ctrl). **b** The relative expression of *miR-708* in 20 pairs of CRC samples and adjacent normal samples by qPCR analyses. **c** The relative expression of *miR-708* in tissues with different disease stages of CRC from TCGA dataset. **d** RNA-FISH assay was performed to detect the level and location of *miR-708* in colonic adenoma from *Apc*^*min*^ mouse (22 weeks old). **e**, **f** The relative expression of *miR-708* in colonic tissues (**e**) and plasma (**f**) from *Apc*^*min*^ mice of the indicated ages. **g**–**i** Plots showing the correlation between *miR-708* and *Meg3* levels in human CRC tissues (**g**), *Apc*^*min*^ mouse colonic tissues (**h**), and CRC cell lines (**i**). The results are presented as the mean ± S.D. in panels (**b**, **c**, **e**, **f**). **p < 0.01, ***p < 0.001 (non-paired two-tailed Student’s *t* test).

Colon cancer might arise from a rare population of cells with stem-cell-like properties. Previous work using the novel *Cre*^*+*^ *Apc*^*+/+*^ mouse model showed that the acute and wide-spread loss of Apc in the colon results in crypt hyperplasia, demonstrating that Apc-deficient cells maintain a “crypt progenitor-like” phenotype [22]. We suspected that abnormal expression of the lncRNA *Meg3* might play a role in stem cell proliferation/transformation. We confirmed our hypothesis using colonic crypts isolated from *Apc*^*min*^ mice, a typical colonic adenoma mouse model [23], and WT mice, showing that *Meg3* accumulates to markedly lower levels in the crypts of colonic tumor samples compared to those from WT mice (Supplementary Fig. 1a). To understand the ceRNA role of *Meg3* in early stage CRC, *Apc*^*min*^ mice were injected with adenovirus-Meg3 (Adv-Meg3) or a control virus (Adv-ctrl), followed by a miR-omes microarray assay. As shown in the heat map in Fig. 1a, the levels of multiple miRNAs were significantly decreased (fold change ≥ 2, *p* value ≤ 0.05) in Adv-Meg3-transformed (*Meg3*-overexpressing) colonic crypts compared to those transformed with the control virus. Among these altered miRNAs, *miR-708* was among the top three miRNAs with decreased accumulation, suggesting that this miRNA may contribute to CRC oncogenesis. *miR-708-5p* has been widely studied for its role as an oncogenic miRNA [24]. Therefore, we next assessed *miR-708* expression in 20 pairs of clinical samples, each consisting of CRC and adjacent normal (control) tissues. We found that *miR-708* accumulated significantly higher levels in CRC tissues compared to the adjacent normal tissues (Fig. 1b). To clarify this observation, the *miR-708* levels in CRC tissues from patients with different pathological stages (normal and stages I, II, and III–IV) were further analyzed, revealing that *miR-708* levels exhibited a positive correlation with CRC malignant progression (Fig. 1c). We used the *Apc*^*min*^ mouse model to obtain samples from animals that spontaneously developed intestinal adenomas. An RNA-FISH assay was performed to detect the expression and location of *miR-708* in the resulting colonic adenomas. We observed that *miR-708* localized primarily in the cell cytoplasm and was detected at a higher level in adenomas compared to the adjacent tissues (Fig. 1d). In addition, *miR-708* levels were measured in *Apc*^*min*^ mice of various ages; the data showed that *miR-708* levels increased in colonic tissues and plasma in a time-dependent manner (Fig. 1e, f).

Given that *Meg3* functions as a critical tumor suppressor in cancer [24, 25], the GEO datasets were analyzed to assess *Meg3* expression in CRC. We found that *Meg3* levels were decreased in tumor tissues from CRC patients (Supplementary Fig. 1b), as well as in AOM/DSS-induced CRC mice (Supplementary Fig. 1c), compared to the corresponding control tissues. Consequently, we quantified *Meg3* and *miR-708* levels in CRC tissues and CRC cells. Correlation analysis of the resulting data revealed that *Meg3* levels negatively correlated with those of *miR-708* in CRC (Fig. 1g–i).

Together, our data demonstrated that *Meg3* levels were negatively correlated with *miR-708* levels; notably, higher *miR-708* levels indicated progression to later stages of CRC. Furthermore, these results suggest that *miR-708* may be a target of *Meg3*, a lncRNA that is responsible for crypt hyperplasia.

### miR-708 deficiency decreases the occurrence of colonic adenoma and inhibits colonic organoid growth

**Fig. 2:**
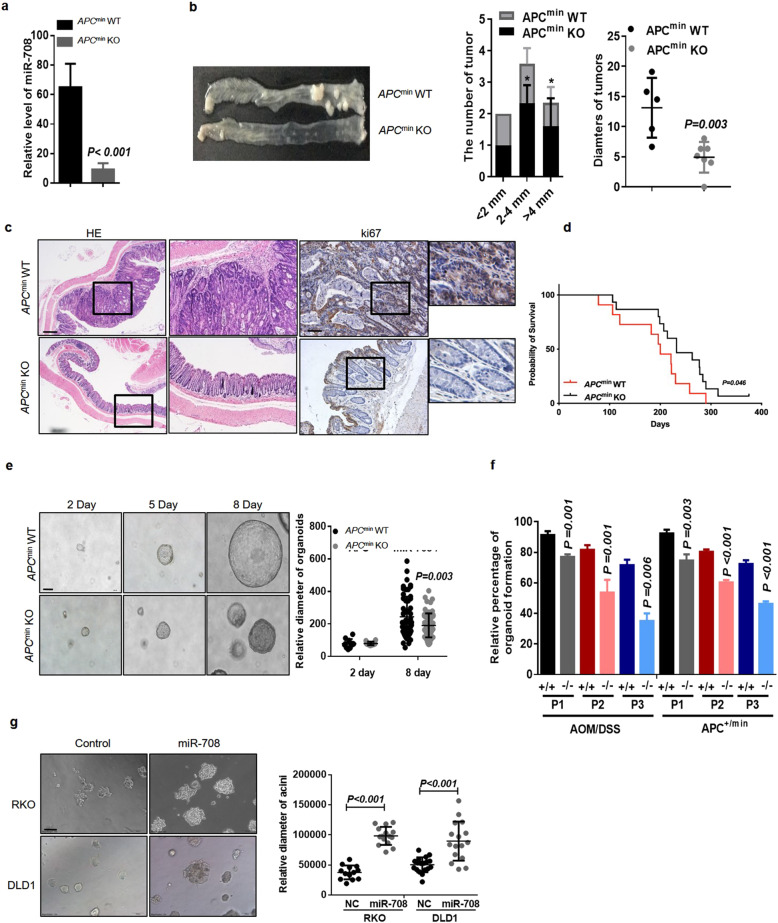
*miR-708* deficiency decreases colonic adenoma formation and inhibits colonic organoid growth. **a**–**d** *Apc*^*min*^ sporadic colorectal cancer model. **a** The knockout effect of *miR-708* in colon tissues from *Apc*^*min*^ mice. **b** Representative images of colonic tumors from *Apc*^*min*^ wild-type (WT) mice and *Apc*^*min*^ *miR-708* KO (KO) mice. The plots show the statistics for tumor number (*y*-axis) at different tumor diameters (*x*-axis). **c** Representative images of HE staining (scale bar: 200 μm) and IHC staining for Ki67 (scale bar: 100 μm). **d** Kaplan–Meier analysis in WT and KO mice. **e** Representative images (scale bar: 100 μm) and quantification of organoid size. Organoids derived from the colonic crypts from *Apc*^*min*^ WT or *Apc*^*min*^ *miR-708* KO mice were cultured for 8 days. **f** The statistics of the percentages of organoid formation after passage (P). Organoids were derived from AOM/DSS-induced colon cancer mice or *Apc*^*min*^ mice. **g** Representative images (scale bar: 100 μm) and quantification of the size of acinar spheres after 6 days of 3D-culture. RKO *miR-708* and DLD1 *miR-708* cells were infected with retrovirus and constitutively express *miR-708*. The results are presented as the mean ± S.D. in panels (**a**, **b**, and **e**–**g**). *p < 0.05 (non-paired two-tailed Student’s *t* test) in panel (**b**).

*miR-708* has been identified as an oncogene in multiple CRC cell lines [25, 26], but evidence for the biological role of *miR-708* in vivo remains limited. Therefore, we generated *miR-708*-knockout *Apc*^*min*^ mice (*Apc*^*min*^ *miR-708*^−/−^) to investigate the role of *miR-708* in early stage CRC. qRT-PCR analysis of *Apc*^*min*^ *miR-708*^−/−^ mice suggested that *miR-708* was depleted successfully in the colon of these animals (Fig. 2a). As shown in Fig. 2b, *Apc*^*min*^ *miR-708*^−/−^ mice exhibited fewer and smaller adenoma tissues compared to *Apc*^*min*^ mice, suggesting *miR-708* depletion significantly (*p* < 0.05) suppresses colon tumor development in vivo (Fig. 2b). It was reported that the intestinal adenomas was appear from 4 weeks in *Apc*^*min*^ mice. Thus, we scarified the *Apc*^*min*^ *miR-708*^+/+^ (wild-type) mice or *Apc*^*min*^ *miR-708*^−/−^ mice at 4 weeks and IHC staining colon tissues for analysis its pathological state. As shown in Fig S2e, there was almost no adenoma burden in the colon of *Apc*^*min*^ *miR-708*^−/−^ (0/6) mice, but we do observe some intestinal adenoma in *Apc*^*min*^*miR-708*^+/+^ (3/7) mice at this stage. Subsequently, we performed staining using H&E and IHC. The results from the H&E staining assay suggested that *miR-708* deficiency attenuated the malignancy of tumor cells, and the data from IHC staining for Ki67 showed that the level of Ki67 was decreased in colonic tissues from *Apc*^*min*^ *miR-708*^−/−^ mice (compared to that in colonic tissues of *Apc*^*min*^ animals), indicating that loss of *miR-708* blocked the over-proliferation of colonic cells typically seen in *Apc*^*min*^ mice (Fig. 2c, Fig S2f). Assessment of the survival of *Apc*^*min*^ mice using Kaplan-Meier analysis revealed that *Apc*^*min*^ *miR-708*^−/−^ mice exhibited better overall survival than *Apc*^*min*^ mice (Fig. 2d). In addition, to further characterize the effect of *miR-708* in early stage CRC, we employed the AOM/DSS-induced CRC model. The results of tumor development, histology, and Ki67 staining in tissues from mice of this model were coincident with those in tissues from *Apc*^*min*^ mice (Supplementary Fig. 2a–f). Together, these data confirmed that *miR-708* has an oncogenic function in early stage CRC.

We established IMCE-Ras cell lines, which contain the activated v-H-ras gene (Fig. S1d). We performed a soft agar assay with the treatment of miR-708 mimic or control mimic in IMCE-Ras cells. Mir-708 mimic treated IMCE-Ras cells have a more spindle-like appearance of transformed cells compared with control treatment (Fig. S1e). Next, to explore the effect of *miR-708* in colonic stem cells, we employed a colonic stem cell-derived organoid culture system (Fig. S1f), in which colonic crypts from *Apc*^*min*^ mice were isolated and then allowed to develop into colonic organoids in Matrigel; this culturing system permitted organoids to maintain physiological characteristics similar to those generated in vivo. We observed that *miR-708* depletion inhibited organoid growth (Fig. 2e), a result consistent with the results obtained with colonic organoids derived from AOM/DSS-induced CRC mice (Supplementary Fig. 2d). Notably, the crypts from *Apc*^*min*^ mice readily developed organoids more frequently than did those from *Apc*^*min*^ *miR-708*^−/−^ mice, and *miR-708* deficiency attenuated the development of organoids after serial passage (Fig. 2f). In addition, our results from 3D- and 2D-culture systems further showed that forced expression of *miR-708* promoted cell proliferation in the RKO and DLD1 CRC cell lines (Fig. 2g). To test the effect of *miR-708* depletion, we designed a *miR-708* antagomir system. As expected, *miR-708* knockdown (using this antagomir) inhibited proliferation in both CRC cell lines (Supplementary Fig. 3a, b).

Taken together, these data suggested that *miR-708* fosters early stage CRC development; this effect may be mediated by elevating the malignant proliferation of colonic stem cells.

### Meg3 sponges miR-708 to inhibit malignant proliferation of colonic stem cells in CRC

**Fig. 3:**
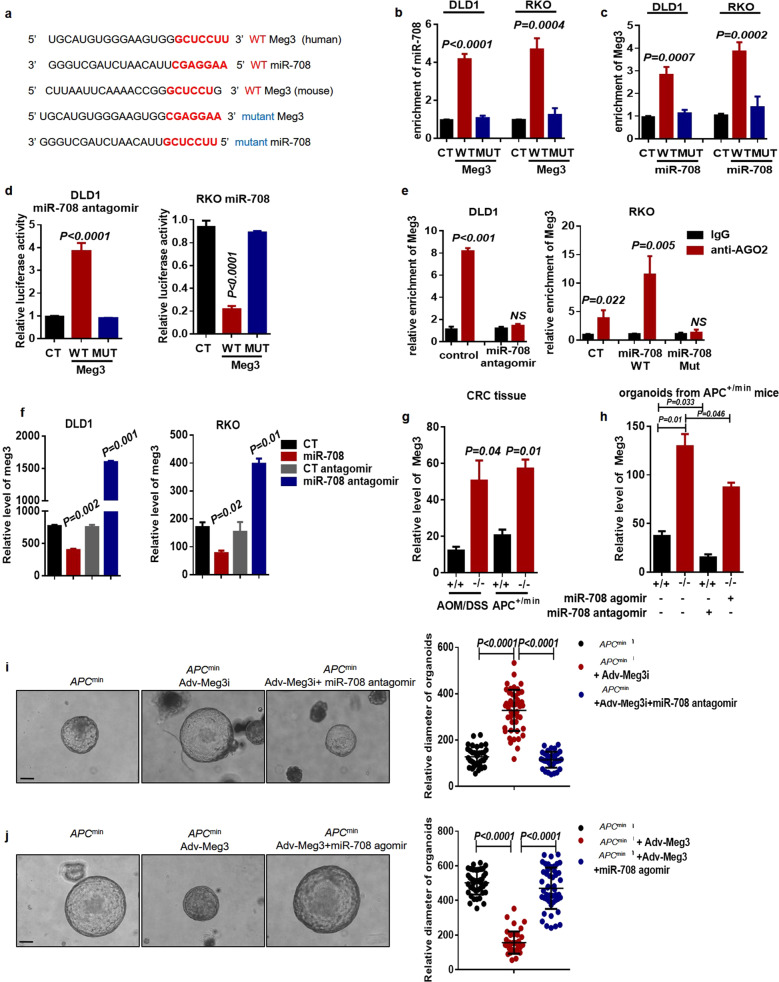
*Meg3* sponges *miR-708* to inhibit malignant proliferation of colonic stem cells in CRC. **a** Predicted base-pairing between *miR-708* and *Meg3* in human and mouse. Mutants (MUT) of *miR-708* and *Meg3* were generated by site-specific mutation of the paired bases. **b** DLD1 and RKO cell lysates were incubated with biotin-labeled *Meg3* (WT and MUT types). RT-qPCR analyses were performed to detect *miR-708* levels in the RNA pulldown obtained using biotin-labeled *Meg3*. **c** DLD1 and RKO cell lysates were incubated with biotin-labeled *miR-708* (WT and MUT types). RT-qPCR analyses were performed to detect *Meg3* levels in the RNA pulldown obtained using biotin-labeled *miR-708*. **d** Luciferase activities were measured in DLD1 and RKO cells. The cells were transfected with the indicated plasmids. **e** An AGO-RIP assay was used to measure *Meg3* levels following *miR-708* knockdown in DLD1 cells (left) or overexpression in RKO cells (right). **f** The relative levels of *Meg3* were detected by qPCR in DLD1 and RKO cells constitutively expressing *miR-708* or in DLD1 and RKO cells transfected with *miR-708* antagomir. **g** RT-qPCR analyses were performed to detect the relative levels of *Meg3* in colonic cancer tissues from AOM/DSS-induced WT (+/+) and *miR-708* KO (−/−) mice or from *Apc*^*min*^ WT (+/+) mice and *Apc*^*min*^ *miR-708* KO (−/−) mice. **h** RT-qPCR analyses were performed to detect the relative levels of *Meg3* in organoids from *Apc*^*min*^ WT (+/+) mice or *Apc*^*min*^ *miR708* KO (−/−) mice. Organoids were treated with *miR-708* agomir or *miR-708* antagomir and cultured for 6 days. **i** Representative images of organoids, derived from *Apc*^*min*^ mice, after 6 days of culturing (scale bar: 100 μm). *Meg3* interference adenovirus (Adv-Meg3i) or control adenovirus was administered to organoids that had been transfected with *miR-708* antagomir or control antagomir. **j** Representative images of organoids, derived from *Apc*^*min*^ mice, after 8 days of culturing (scale bar: 100 μm). *Meg3* overexpression adenovirus (Adv-Meg3) or control adenovirus was administered to organoids that had been transfected with *miR-708* agomir or control agomir. The results are presented as the mean ± S.D. in panels (**b**–**j**). NS not significant (non-paired two-tailed Student’s *t* test).

Given that *miR-708* levels are decreased in the presence of *Meg3* in colonic crypt cells, we hypothesized that *Meg3* acts as a ceRNA in regulating *miR-708* levels, which led us to investigate the potential sponging relationship between *Meg3* and *miR-708*. To address the underlying mechanism, database prediction tools were used. Bioinformatics analyses identified putative *Meg3*-binding sequences in *miR-708* and showed that these putative binding sequences are conserved between humans and mice (Fig. 3a). Next, we constructed *Meg3*- and *miR-708*-WT or Mut plasmids based on these potential binding sequences; the plasmids were labeled with biotin and then transiently transfected into DLD1 and RKO cell lines (respectively), followed by the enrichment detection using an RNA pull-down assay. The results showed that *miR-708* was enriched in *Meg3*-WT-pulled down RNAs, while *Meg3*-Mut failed to pull down *miR-708* (Fig. 3b). Conversely, *Meg3* was pulled down by *miR-708*-WT but not by *miR-708*-Mut (Fig. 3c). These data suggested that *Meg3* specifically binds to *miR-708* by base pairing. To further verify the *Meg3*-*miR-708* interaction, a luciferase reporter assay was performed by co-transfecting CRC cell lines either with pGL3-Meg3-WT in combination with the *miR-708* agomir or with pGL3-Meg3-Mut in combination with the *miR-708* antagomir. We found that *miR-708* negatively regulated luciferase activity only with *Meg3*-WT, and not with *Meg3*-Mut (Fig. 3d). Anti-AGO2 RIP assays also have been used to measure the endogenous interactions between a miRNA and its targeted sequences, given that AGO2 is critical for miRNA-induced RNA repression and degradation [27]. Therefore, we conducted an anti-AGO2 RIP assay in CRC cancer cells. The results showed that *miR-708* counteracted the accumulation of *Meg3*, while a *miR-708* antagomir had the opposing effect (Fig. 3e). Collectively, these results indicated the *Meg3* interacts with *miR-708* and is involved in a *miR708*-mediated RNA silencing complex.

To further validate the negative correlation between *Meg3* and *miR-708* levels in CRC cells, we detected *Meg3* levels in CRC cells with forced expression of *miR-708* or that had been transfected with a *miR-708* antagomir. In both cell lines, qRT-PCR analysis showed that *miR-708* overexpression counteracted *Meg3* accumulation in DLD1 and RKO cells, whereas introduction of the *miR-708* antagomir led to *Meg3* accumulation (Fig. 3f). Furthermore, in either *Apc*^*min*^ mice or AOM/DSS-treated mice, *miR-708* deficiency elevated *Meg3* levels in colonic tissues as well as in colonic crypt cells (Fig. 3g, h). We further explored the role of the *Meg3*-*miR-708* axis in colonic stem cell growth using the organoid culture system and found that loss of *Meg3* enhanced organoid growth, while overexpression of *Meg3* suppressed colonic organoid growth (Fig. 3i, j). As expected, the *miR-708* agomir rescued the *Meg3*-induced growth inhibition; consistent with that observation, the *miR-708* antagomir counteracted the increased organoid growth seen with *Meg3* deficiency (Fig. 3i, j). In addition, the data from the proliferation assay verified that *Meg3* inhibited CRC cell proliferation, an effect that was reversed by *miR-708* (Supplementary Fig. 4).

Together, these results demonstrated that *Meg3* inhibits malignant proliferation of colonic stem cells and CRC cells via sponging of *miR-708*.

### miR-708 promotes malignant proliferation of colonic cells via targeting of SOCS3 expression

**Fig. 4:**
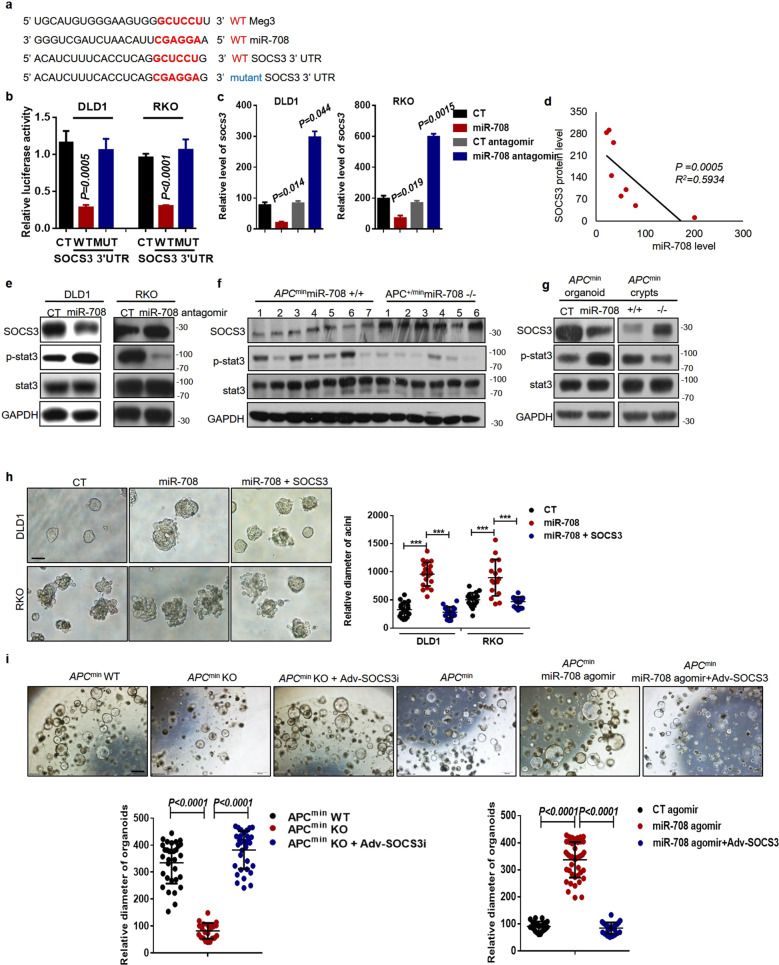
*miR-708* promotes malignant proliferation of colonic cells via targeting of SOCS3 expression. **a** Predicted base-pairing between *SOCS3* 3′-UTR and *miR-708*. The mutant type (MUT) of the *SOCS3* 3′-UTR was generated by site-specific mutation of the paired bases. **b** The relative luciferase activities in DLD1 and RKO cells that were transfected with *miR-708* and *SOCS3* WT 3′UTR or *SOCS3* MUT 3′UTR. **c** RT-qPCR analyses were performed to measure *SOCS3* mRNA levels in DLD1 (left) and RKO (right) cells constitutively expressing *miR-708*, or in DLD1 and RKO cells transfected with a *miR-708* antagomir. **d** The statistics of *miR-708* levels and SOCS3 protein levels in eight colorectal cancer cell lines. **e** Western blotting was performed to detect levels of SOCS3 and activated STAT3 in RKO cells with knockdown of *miR-708* and in DLD1 cells with forced expression of *miR-708*. **f** Western blotting was performed in colonic tumor tissues from *Apc*^*min*^ WT mice and from *Apc*^*min*^ KO mice. **g** Western blotting was performed in organoids. Organoids derived from *Apc*^*min*^ mice were treated with control agomir (CT) or *miR-708* agomir (*miR-708*) and cultured for 6 days; organoids from *Apc*^*min*^ WT mice (+/+) and *Apc*^*min*^ *miR-708* KO mice (−/−) were cultured for 6 days. **h** Representative images (scale bar: 100 μm) and quantification of the size of acinar sphere after 6 days of 3D-culture. Cells constitutively expressing *miR-708* had been transfected with pCMV-SOCS3. **i** Representative images (scale bar: 100 μm) and quantification of the size of organoids from *Apc*^*min*^ mice. Organoids, derived from *Apc*^*min*^ WT mice and from *Apc*^*min*^ *miR-708* KO mice, were infected with Adv-control or Adv-SOCS3 and cultured for 7 days; organoids derived from *Apc*^*min*^ mice were treated with *miR-708* agomir or control agomir and Adv-control or Adv-SOCS3 and were cultured for 7 days. The results are presented as the mean ± S.D. in panels (**b**, **c**, **h**, **i**). Statistical analyses were conducted by non-paired two-tailed Student’s *t* tests.

Previous studies have suggested that SOCS3 inhibits CRC development and is indicative of a better prognosis in CRC patients [28, 29]. Interestingly, bioinformatics analyses by several targeted mRNA-prediction tools have revealed the presence of *miR-708*-complementary sequences in the *SOCS3* 3′UTR; notably, these putative binding sequences are the same as those shared between *Meg3* and *miR-708* (Fig. 4a). Herein, we performed luciferase reporter assays to confirm the targeting relationship and found that expression of *miR-708*-WT led to a significant decrease in luciferase activity in CRC cell lines, whereas the luciferase activity in cells overexpressing *miR-708*-Mut was comparable to that in control cells (Fig. 4b). Consistently, forced expression of *miR-708* was associated with decreased levels of *SOCS3* mRNA and protein, while the introduction of a *miR-708* antagomir led to the accumulation of *SOCS3* mRNA and protein (Fig. 4c, e). In addition, a negative correlation between *miR-708* and SOCS3 protein levels was observed in CRC cell lines (Fig. 4d).

Given that SOCS3 acts as a negative regulator of the JAK/STAT3 pathway [30, 31], we next assessed STAT3 activation. Western blotting and IF staining suggested that *miR-708* overexpression elevates STAT3 activation in DLD1 cells, an observation that was further supported by the data from analyses of the expression of STAT3-targeted genes (Fig. 4e, Supplementary Fig. 5a, b). In addition, *miR-708* deficiency led to attenuation of STAT3 activation via elevation of SOCS3 expression in colonic tissues from *Apc*^*min*^ mice as well as in colonic crypt cells from *Apc*^*min*^ mice (Fig. 4f, g). Consistent with those results, a *miR-708* agomir inhibited SOCS3 accumulation, with associated potentiation of STAT3 activation in colonic organoids derived from crypts isolated from *Apc*^*min*^ mice (Fig. 4g). Furthermore, IHC staining indicated that the level of p-STAT3 was decreased in AOM/DSS-induced *miR-708* KO mice, in contrast to the effect in control mice (Supplementary Fig. 5c). Together, these data suggested that *miR-708* activates STAT3 through antagonism of SOCS3 accumulation in CRC.

To further explore the effect of *miR-708*/SOCS3/STAT3 in the malignant proliferation of CRC cells, an adenovirus driving SOCS3 overexpression was used in both the 2D- and 3D-culturing systems. The results showed that SOCS3 overexpression blocked *miR-708*-fostered CRC cell proliferation in both culture systems (Fig. 4h, Supplementary Fig. 5d). Notably, the malignant proliferation of colonic crypt cells from *Apc*^*min*^ mice with abundant *miR-708* was markedly dampened by SOCS3; in contrast, SOCS3 knockdown reversed the inhibition of colonic organoid growth caused by loss of *miR-708* in colonic crypts from *Apc*^*min*^ mice (Fig. 4i).

Taken together, our results indicated that *miR-708* promotes STAT3 activation by targeting the *SOCS3* 3′-UTR, resulting in depletion of SOCS3 levels and causing malignant proliferation of colonic stem cells and CRC cell lines.

### Meg3 protects SOCS3 from miR-708-induced depletion

**Fig. 5:**
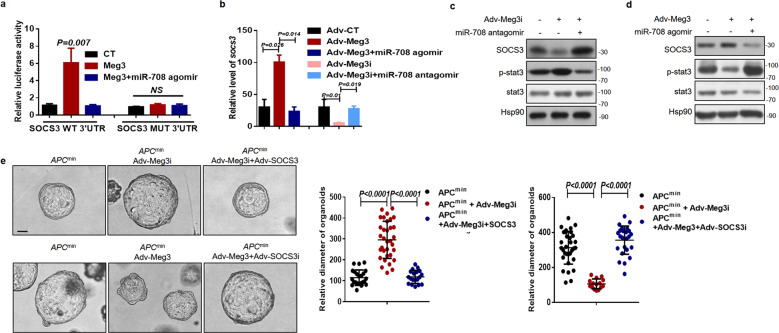
*Meg3* protects SOCS3 from *miR-708*-induced depletion. **a** The relative luciferase activities in RKO cells that had been transfected with indicated constructs. **b** Relative levels of *Meg3* were detected by qPCR in RKO cells that had been infected with indicated adenovirus (to overexpress or knockdown *Meg3*) and then treated with *miR-708* agomir or *miR-708* antagomir, respectively. **c** Western blotting was performed to measure SOCS3 and activated STAT3 in DLD1 cells. Cells were infected with Adv-Meg3i, followed by treatment with *miR-708* antagomir or control antagomir. **d** Western blotting was performed to measure SOCS3 and activated STAT3 in RKO cells. Cells were infected with Adv-Meg3, followed by treatment with *miR-708* agomir or control agomir. **e** Representative images (scale bar: 100 μm) and quantification of organoid size. Upper: Organoids derived from *Apc*^+/^^*min*^ mice were infected with Adv-control, Adv-Meg3i, or Adv-Meg3i + Adv-SOCS3, or infected with Adv-control, Adv-Meg3, or Adv-Meg3 + Adv-SOCS3i. The results are presented as the mean ± S.D. in panels (**a**, **b**, and **e**). NS not significant (non-paired two-tailed Student’s *t* test).

Next, to determine the critical role of *Meg3* in the *miR-708*/SOCS3 axis, we used luciferase reporter assays in RKO cells to show that *Meg3* overexpression increased the luciferase activity of a *SOCS3*-WT-containing construct; this effect was counteracted by the introduction of a *miR-708* agomir (Fig. 5a). As expected, infection of DLD1 cells with the adenovirus-*Meg3* interference construct resulted in a decrease in luciferase activity, which was restored by the introduction of a *miR-708* antagomir (Supplementary Fig. 6a). qRT-PCR and western blot assays then were used to measure the potential regulation of SOCS3 expression by *Meg3*. The results showed that *Meg3* overexpression enhanced *SOCS3* mRNA and protein levels in RKO cells, while knockdown of *Meg3* attenuated *SOCS3* expression in DLD1 cells (Fig. 5b–d). However, the *Meg3*-induced accumulation of SOCS3 was blocked by a *miR-708* agomir (Fig. 5b, d). Furthermore, the expression of *Meg3* and SOCS3 were analyzed in various CRC cell lines, revealing that *Meg3* levels positively correlated with those of the SOCS3 protein (Supplementary Fig. 6b). Subsequently, activation of STAT3 was tested. The results suggested that *Meg3* negatively regulated STAT3 activation and the expression of downstream target genes; those effects were reversed by a *miR-708* agomir (Fig. 5b-d, Supplementary Fig. 6c, d). Thus, our data suggested that *Meg3* elevates SOCS3 levels by sponging *miR-708* in CRC.

Functionally, the malignant proliferation of colonic stem cells was observed in the organoid culture system. Meg3i and SOCS3i adenoviruses were used to infect arganoids and in vitro cell culture (Supplementary Fig. 1g, h). We found that SOCS3 overexpression increased growth in Adv-Meg3i-infected organoids derived from the colonic crypts of *Apc*^*min*^ mice. In contrast, SOCS3 deficiency decreased the growth of these organoids when the cells were infected with Adv-Meg3 (Fig. 5e).

Together, these data revealed that *Meg3*-inhibited malignant proliferation in CRC is mediated by *miR-708*/SOCS3 signaling.

### Meg3/miR-708/SOCS3 pathway is associated with CRC development

**Fig. 6:**
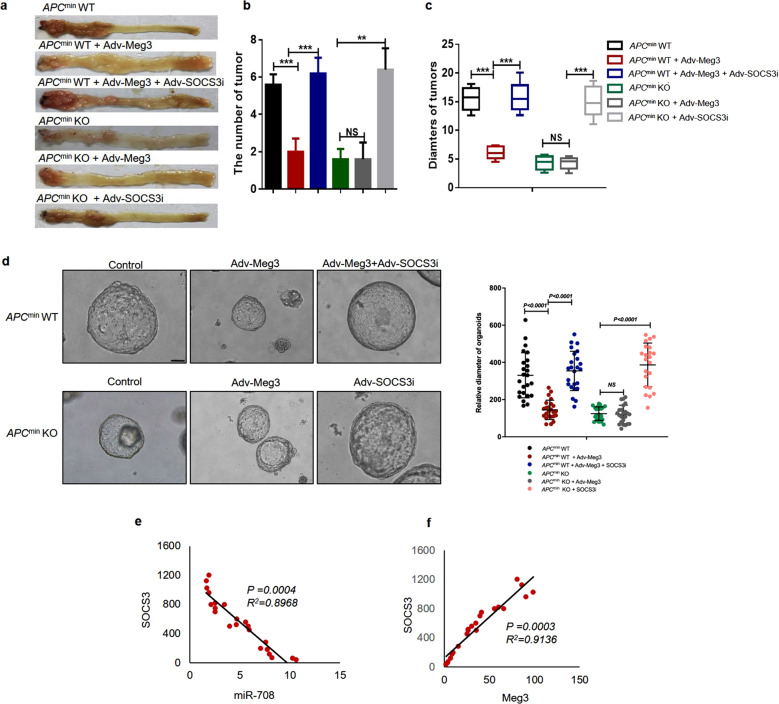
The *Meg3*/*miR-708*/SOCS3 pathway is closely associated with CRC development. **a**–**c** *Apc*^*min*^ WT mice or *Apc*^*min*^ KO mice were infected with Adv-Meg3, Adv-SOCS3i, or Adv-Meg3 + Adv-SOCS3i by coloclysis. **a** Representative images of colonic tumors from *Apc*^*min*^ mice subjected to the indicated treatments. **b** Plot showing the numbers of tumors (diameter: 2–4 mm) from *Apc*^*min*^ mice subjected to the indicated treatments. **c** The statistics of the diameters of tumors from *Apc*^*min*^ mice subjected to the indicated treatments. **d** Typical images (scale bar: 100 μm) of organoids derived from the mice presented in (**a**). **e**, **f** Plots showing the correlation between *miR-708*, *Meg3*, and SOCS3 levels in CRC tissues (*N* = 22). The results are presented as the mean ± S.D. in panels (**b** and **c**). NS not significant (non-paired two-tailed Student’s *t* test).

To further confirm the function of the *Meg3*/*miR-708*/SOCS3 axis in CRC development, we altered the expression of *Meg3* and SOCS3, either alone or together, in the colons of *Apc*^*min*^ *miR-708*^−/−^ mice or *Apc*^*min*^ mice using adenovirus-mediated infection in vivo. We observed that *Meg3* overexpression inhibited tumor growth in *Apc*^*min*^ mice, rendering a phenotype similar to that of *Apc*^*min*^ *miR-708*^−/−^ mice (Fig. 6a–c). These results confirmed that *Meg3* acts as a tumor suppressor in CRC, whereas *miR-708* acts as an oncogene in CRC. However, the number and weight of tumors were increased in *Apc*^*min*^ mice infected with Adv-SOCS3i + Adv-Meg3 (compared to *Apc*^*min*^ mice infected with control constructs). In addition, interference in SOCS3 expression in the colon promoted tumor growth in *Apc*^*min*^ *miR-708*^−/−^ mice (Fig. 6a–c), indicating that the blocking effect of *miR-708* deficiency on tumor growth was reversed by loss of SOCS3. Thus, SOCS3 is an effector of the *Meg3*/*miR-708* signaling axis in vivo.

In further experiments, we cultured tumor organoids derived from CRC tissues of patients. We found that forced expression of *Meg3* inhibited the growth of these organoids, while loss of SOCS3 counteracted the effect of *Meg3* on organoid growth (Fig. 6d). Subsequently, we examined the clinical significance of the findings established in our murine model. Analyses of the expression levels of *Meg3*, *miR-708*, and SOCS3 in human CRC tissues showed that *Meg3* levels correlated positively with SOCS3 levels, while *miR-708* levels correlated negatively with SOCS3 levels (Fig. 6e, f). Notably, these results in clinical samples paralleled the data obtained with CRC cell lines. Thus, the *Meg3*/*miR-708*/SOCS3 signaling axis is relevant in human CRC.

Collectively, these results demonstrated that the *Meg3*/*miR-708*/SOCS3 signaling axis exists in CRC.

## Discussion

In the present study, we uncovered, for the first time (to our knowledge), the underlying mechanism by which *Meg3* inhibits the malignant proliferation of colonic stem cells in early stage CRC. *Meg3* levels negatively correlated with *miR-708* levels, both in vivo and in vitro, particularly in colonic crypts from subjects with CRC. In addition, *miR-708* deficiency attenuated colon tumor development and colonic organoid growth via direct targeting of SOCS3 accumulation. Importantly, *Meg3* acts as a ceRNA for *miR-708*, leading to enhanced expression of SOCS3, which in turn suppresses STAT3 signaling and malignant proliferation of colonic stem cells during the early stage of colon tumor formation. In the clinic, the high *miR-708* expression has been shown to indicate poor prognosis in patients with CRC. Based on these data, we hypothesize that *Meg3* functions as a ceRNA to regulate SCOS3 expression through sponging of *miR-708*, thereby exerting regulatory functions to inhibit the malignant proliferation of colonic stem cells in early stage CRC. Thus, *Meg3* may be a promising target for diagnosis and therapy of early stage CRC.

The lncRNA *Meg3*, which is abundantly expressed in many normal tissues, plays a central role in development and growth [32]. However, increasing evidence suggests that *Meg3* frequently is either lost or depleted in multiple human tumors and tumor cell lines [11–13]. Clinically, *Meg3* levels strongly correlated with the clinicopathological outcomes of various cancers [12]. In particular, lower levels of *Meg3* are associated with worse pathological grade and advanced pathological TNM stage in CRC [14], an observation that is consistent with the results of the present study. Interestingly, our data revealed, for the first time, that *Meg3* serves as a critical inhibitor of the malignant proliferation of colonic stem cells, the cell type that is mainly responsible for the majority of CRC development, especially in early stage CRC. Given the significant role of pathological stage at diagnosis in the clinic, *Meg3* might be a promising candidate for application in the diagnosis of early stage CRC.

lncRNA, by serving as ceRNAs, are known to regulate transcript accumulation at the post-transcriptional level by competing for miRNAs harboring complementary sequences [9]. *Meg3* exhibits its biological roles via miRNAs that have been identified based on their roles in cancer, including (for example) *miR-141* in chemotherapy [33], *miR-212* in EMT [34], *miR-19a* and *miR-93* in the cell cycle [35, 36], and *miR-21-5p* in cell apoptosis [37]. In contrast, there have been (to our knowledge) only a limited number of studies related to the potential role of a *Meg3*/miRNA axis in CRC. In the present study, we revealed that *Meg3* acts as ceRNA to sponge *miR-708* in early stage CRC. Several studies have confirmed that *miR-708* acts as an oncogenic miRNA via regulation of proliferation in CRC cell lines, as verified in the present work. Our results further showed that *miR-708* accumulates to high-grade in tumor tissues from patients with CRC, such that the *miR-708* levels positively correlated with CRC progression. Importantly, we also showed, using mouse models and organoid culture systems, that *miR-708* is responsible for the malignant proliferation of colonic stem cells and early stage CRC development. Notably, *Meg3* deficiency dramatically attenuated tumor growth in *Apc*^*min*^ *miR-708*^−/−^ mice, hinting that the combined application of agomirs of *Meg3* and antagomirs of *miR-708* might contribute to therapy of CRC in the clinic.

Our analysis also demonstrated that regulation of mRNA accumulation is involved in events downstream of this lncRNA/miRNA signaling axis. Our bioinformatics analyses and luciferase assays verified *SOCS3* as a potential target mRNA of the *Meg3*/*miR-708* axis. As a critical negative regulator of the JAK/STAT signaling pathway, SOCS3 inhibits proliferation, migration, and invasion by blocking STAT3 activation, which is critical for lymph node metastasis and advanced clinical stages of CRC [28–31, 38]. Interestingly, our data indicated that manipulating miRNA-708 could influence the expression of Meg3. We found that quite a few studies reported that IL6 activates STAT3 to unregulated DNA methyltransferase (DNMT), such as DNMT3b and DNMT expression is important to the tumorigenesis [39–41]. It is known that DNA-demethylating drugs blocked cell proliferation and activated Meg3 expression. We have demonstrated the miRNA-708/SOCS3/p-STAT3 axis in colon tumorigenesis in this study. We guess that miRNA-708 may upregulate Meg3 by evaluation of DNMT3b, which was related to inhibition of SOCS3 and activation of p-STAT3. The present study demonstrated that *Meg3* elevates SOCS3 accumulation by sponging *miR-708*; the resulting increase in SOCS3 accumulation impedes STAT3 activation and finally dampens the malignant proliferation of colonic stem cells in early stage CRC. Intriguingly, previous data suggested that STAT3 regulates *Meg3* expression by binding to the *Meg3* promoter [42]. Thus, a feedback loop appears to exist between *Meg3* and the SOCS3/STAT3 signaling pathway, an insight that deserves further exploration.

In conclusion, the present study demonstrated that *Meg3* sponges *miR-708* to inhibit early stage CRC development; this effect is mediated by SOCS3 inhibition of malignant proliferation of colonic stem cells. Thus, these results provide a novel insight into the mechanism underlying the role of *Meg3* in early stage CRC. Targeting of *Meg3* and *miR-708* might represent a promising strategy in both the diagnosis and therapy of colorectal tumors.

## Supplementary information

Supplementary materials

Figure s1

Figure s2

Figure s3

Figure s4

Figure s5

Figure s6

Reproducibility checklist

## Data Availability

The datasets used and analyzed during the current study are available from the corresponding author on reasonable request.
