# Supplementary material for: Long noncoding RNA Meg3 sponges miR-708 to inhibit intestinal tumorigenesis via SOCS3-repressed cancer stem cells growth
Source: Cell Death Dis. 2021 Dec 21;13(1):25. doi: 10.1038/s41419-021-04470-5 (PMC8692598; doi:10.1038/s41419-021-04470-5)
Supplement: Supplementary file 1 — Supplementary materials [file 41419_2021_4470_MOESM1_ESM.docx]

**Supplementary materials**

Fig. S1. Meg3 levels are decreased in colon tumor tissues. a, Meg3 levels in colonic tissues from C57BL/6 mice and Apc^min^ mice. b, Meg3 levels in colon tumor tissues and paired adjacent normal tissues from the GSE70880 dataset. c, Meg3 levels in colon tissues of AOM/DSS-treated mice from the GSE121128 dataset. d, Western blotting was performed to measure H-Ras in IMCE and IMCE-Ras cell lines. e, The colony formation ability of IMCE-Ras cells which were transfected with miR-708 mimic or control mimic. f, Flow cytometry was used to detect the positive proportion of LGR5 in organoid cells. g ,h, The knockdown efficiency of Adv-Meg3i and Adv-SOCS3i .The results are presented as the mean±S.D. Statistical analyses were conducted using non-paired two-tailed Student’s t-tests.

Fig. S2. mir-708 deficiency suppresses tumor development in AOM/DSS induced colonic tumor model. a-c, AOM/DSS-induced colorectal cancer model. a, The knockout effect of miR-494 in colon tissues from AOM/DSS-treated mice. B, Representative images of colonic tumors in WT and miR-708 KO mice that were subjected to AOM/DSS-induced colon cancer model. The plots show the statistics for tumor number (left) and tumor diameter (right). c, Representative images of HE staining (scale bar: 200 μm) and IHC staining for Ki67 (scale bar: 100 μm). d, Representative images (scale bar: 100 μm) and quantification of organoid size. Organoids derived from the colonic crypts of AOM/DSS-treated WT or miR-708 KO mice were cultured for 6 days. e, The incidence of colonic adenoma in Apc^min^ miR-708-/- (0/6) and Apc^min^ miR-708+/+ (3/7)mice. f. Representative images of HE staining (scale bar: 200 μm) and IHC staining for PCNA (scale bar: 100 μm) in colon tissues from Apc^min^ miR-708-/- and Apc^min^ miR-708+/+ mice. The results are presented as the mean±S.D in panels (b and d). Statistical analyses were conducted using non-paired two-tailed Student’s t-tests.

**Fig. S3.** *miR-708* promotes cell proliferation in CRC cell lines. a, Cell Counting Kit-8 Assays were performed to assess cell growth in DLD1 cells transfected with *miR-708* agomir, *miR-708* antagomir, or control agomir. b, Cell Counting Kit-8 Assays were performed to assess cell growth in RKO cells transfected with *miR-708* agomir, *miR-708* antagomir, or control agomir. The results are presented as the mean±S.D. Statistical analyses were conducted using non-paired two-tailed Student’s t-tests.

**Fig. S4.** *Meg3* inhibits cell proliferation by sponging *miR-708* in CRC cell lines. a, Cell Counting Kit-8 Assays were performed to assess cell growth in RKO cells transfected with *Meg3* or *Meg3* + *miR-708* agomir. b, Cell Counting Kit-8 Assays were performed to assess cell growth in RKO cells infected with Adv-Meg3i or Adv-Meg3i + *miR-708* antagomir. c, *miR-708* levels and *Meg3* levels in eight colorectal cancer cell lines. The results are presented as the mean±S.D. Statistical analyses were conducted using non-paired two-tailed Student’s t-tests.

**Fig. S5.** *miR-708* promotes cell proliferation by targeting SOCS3/STAT3 signaling. a, DLD1 cells cultured in a 3D-culture system were subjected to immunofluorescence (IF) staining for p-STAT3 (scale bar: 50 μm). b, qPCR analyses were performed to detect the levels of target genes of STAT3 signaling in DLD1 cells that had been transfected with *miR-708* agomir, *miR-708* antagomir, or control agomir. c, IHC staining for SOCS3 and p-STAT3 in colon tissues from AOM/DSS-treated WT or KO mice (scale bar: 100μm). d, Cell Counting Kit-8 Assays were performed to assess cell growth in DLD1 and RKO cells. Cells constitutively expressing *miR-494* were transfected with pCMV-SOCS3. The results are presented as the mean±S.D. in panels (b and d). Statistical analyses were conducted using non-paired two-tailed Student’s t-tests.

**Fig. S6.** *Meg3* protects SOCS3 from *miR-708*-targeted decrease though competitive interaction with *miR-708*. a, The relative luciferase activities in DLD1 cells infected with or without Adv-Meg3i and then transfected with *miR-708* antagomir or control antagomir. b, *Meg3* levels and SOCS3 protein levels in eight colorectal cancer cell lines. c, qPCR analyses were performed to detect the levels of target genes of STAT3 signaling in RKO cells infected with or without Adv-Meg3 and then transfected with *miR-708* agomir or control agomir. d, qPCR analyses were performed to detect the levels of target genes of STAT3 signaling in DLD1 cells infected with or without Adv-Meg3i and then transfected with *miR-708* antagomir or control antagomir. The results are presented as the mean±S.D. in panels (a, c and d). *NS*, not significant. Statistical analyses were conducted using non-paired two-tailed Student’s t-tests.
