# Supplementary figures and images for: Long noncoding RNA Meg3 sponges miR-708 to inhibit intestinal tumorigenesis via SOCS3-repressed cancer stem cells growth
Source: Cell Death Dis. 2021 Dec 21;13(1):25. doi: 10.1038/s41419-021-04470-5 (PMC8692598; doi:10.1038/s41419-021-04470-5)

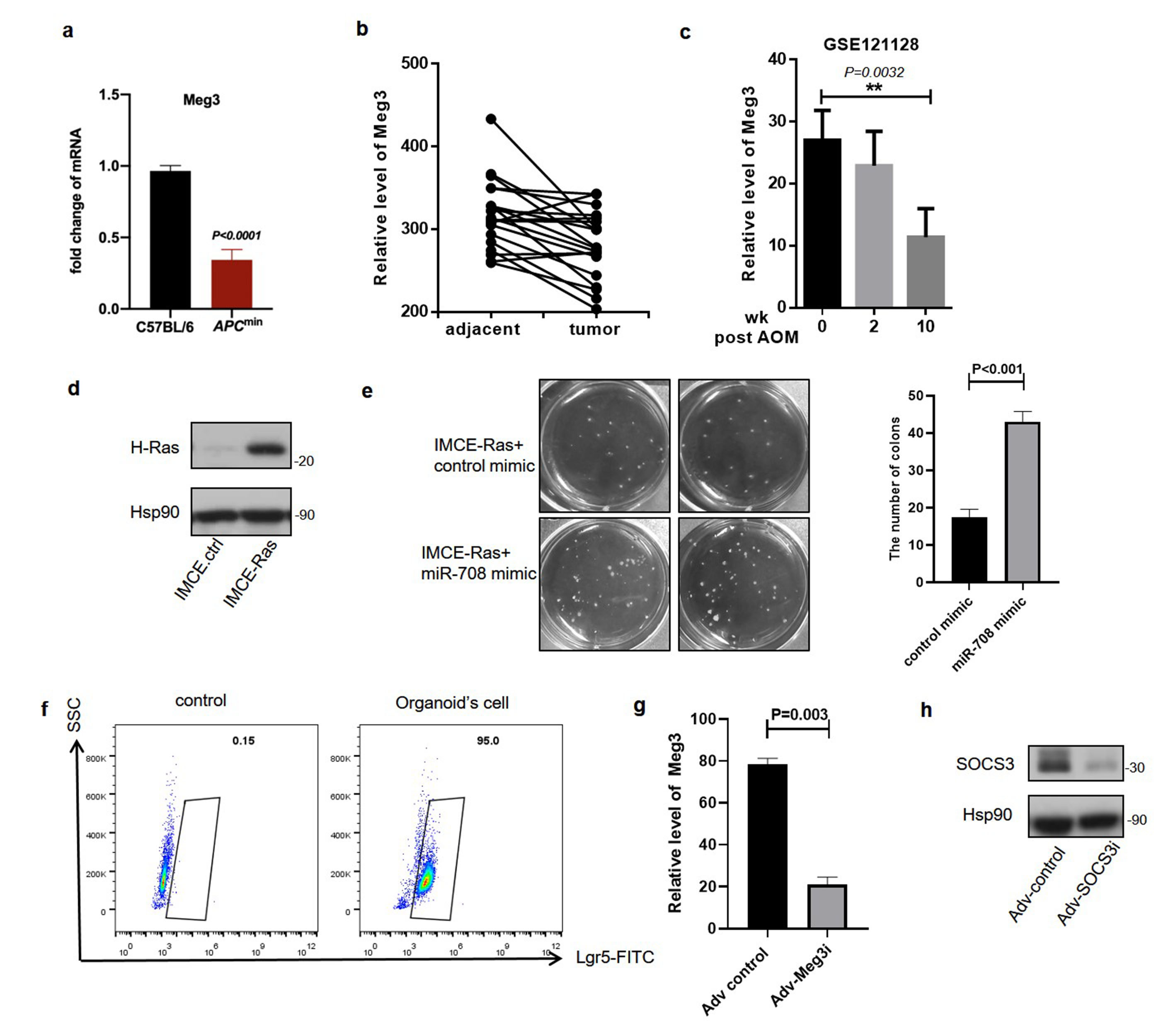

Supplement: Supplementary file 2 — Figure s1 [file 41419_2021_4470_MOESM2_ESM.tif]

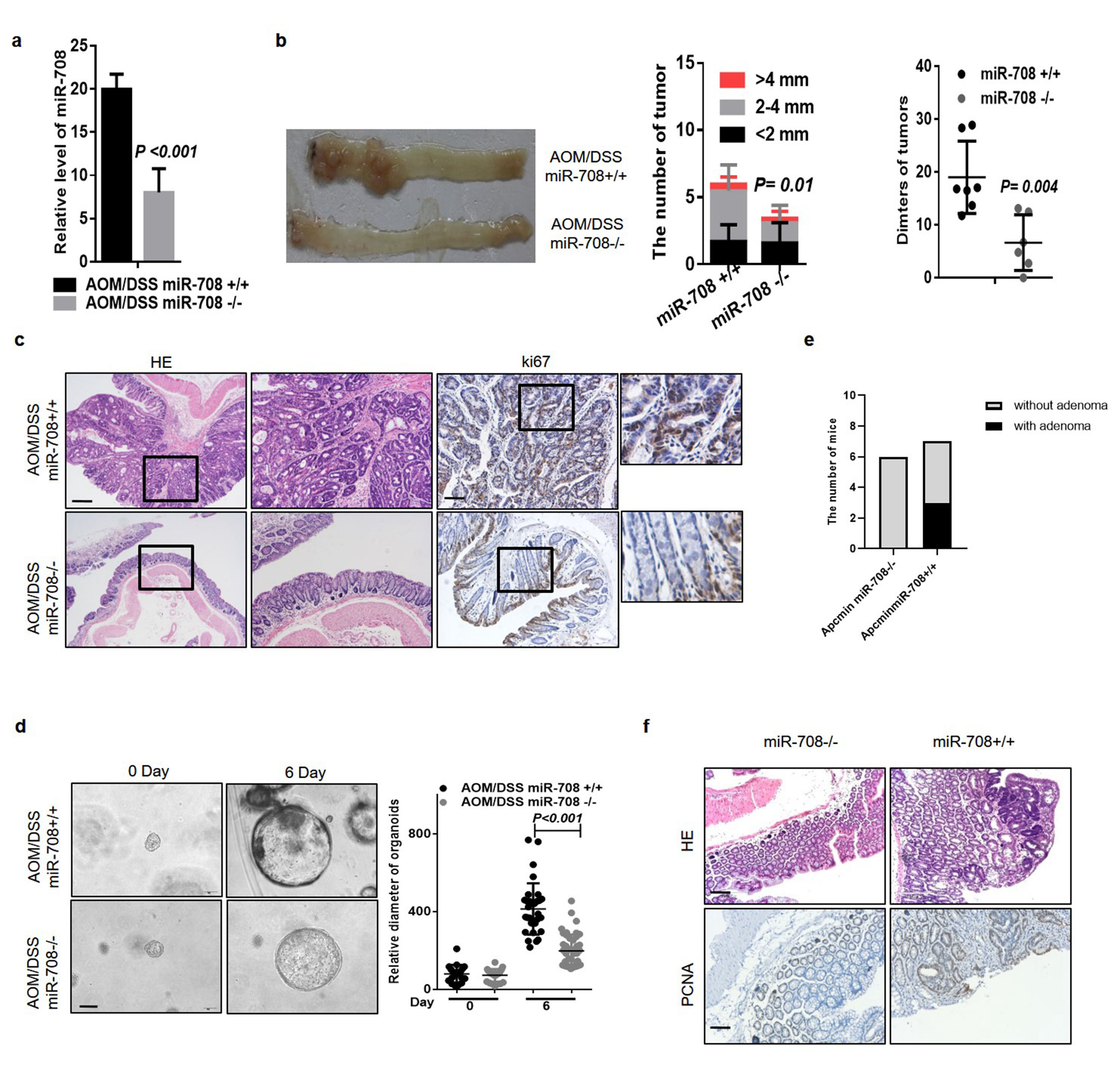

Supplement: Supplementary file 3 — Figure s2 [file 41419_2021_4470_MOESM3_ESM.tif]

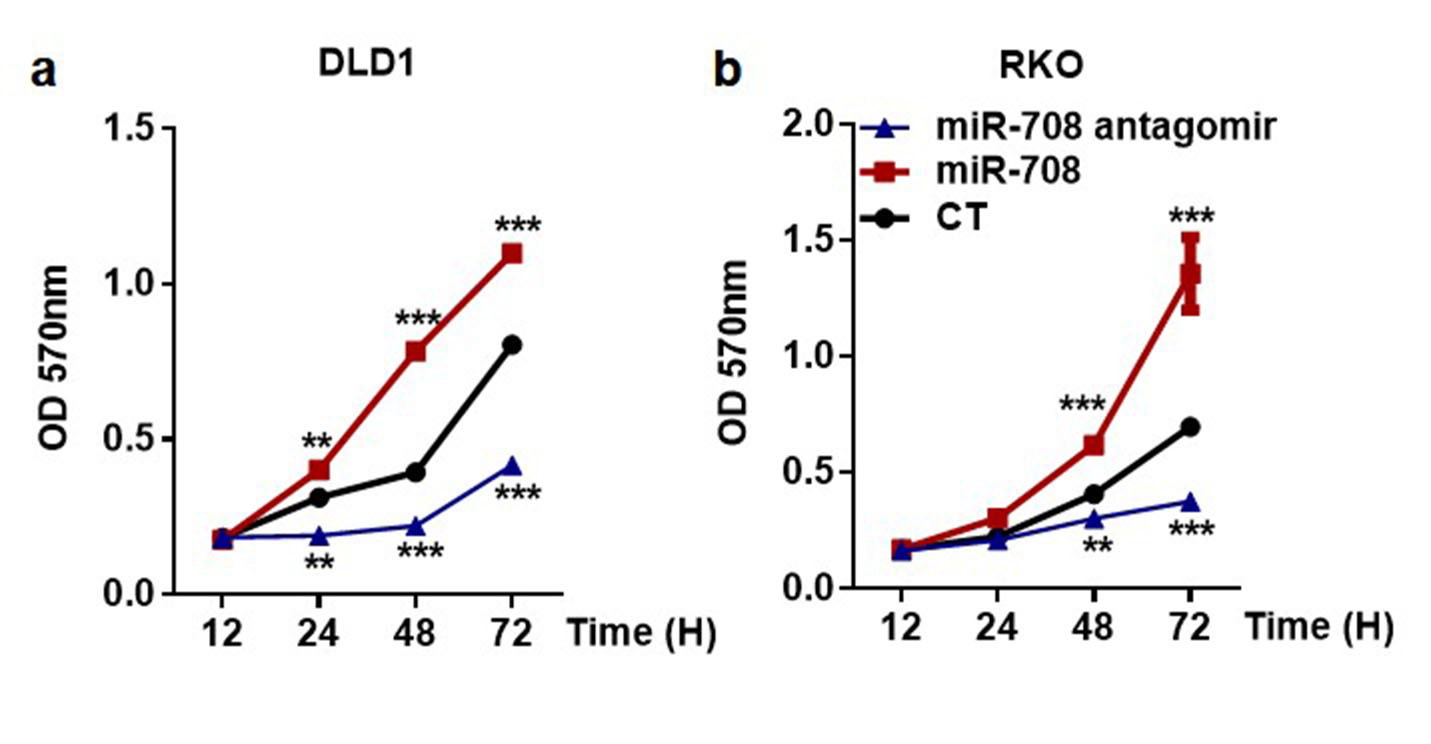

Supplement: Supplementary file 4 — Figure s3 [file 41419_2021_4470_MOESM4_ESM.tif]

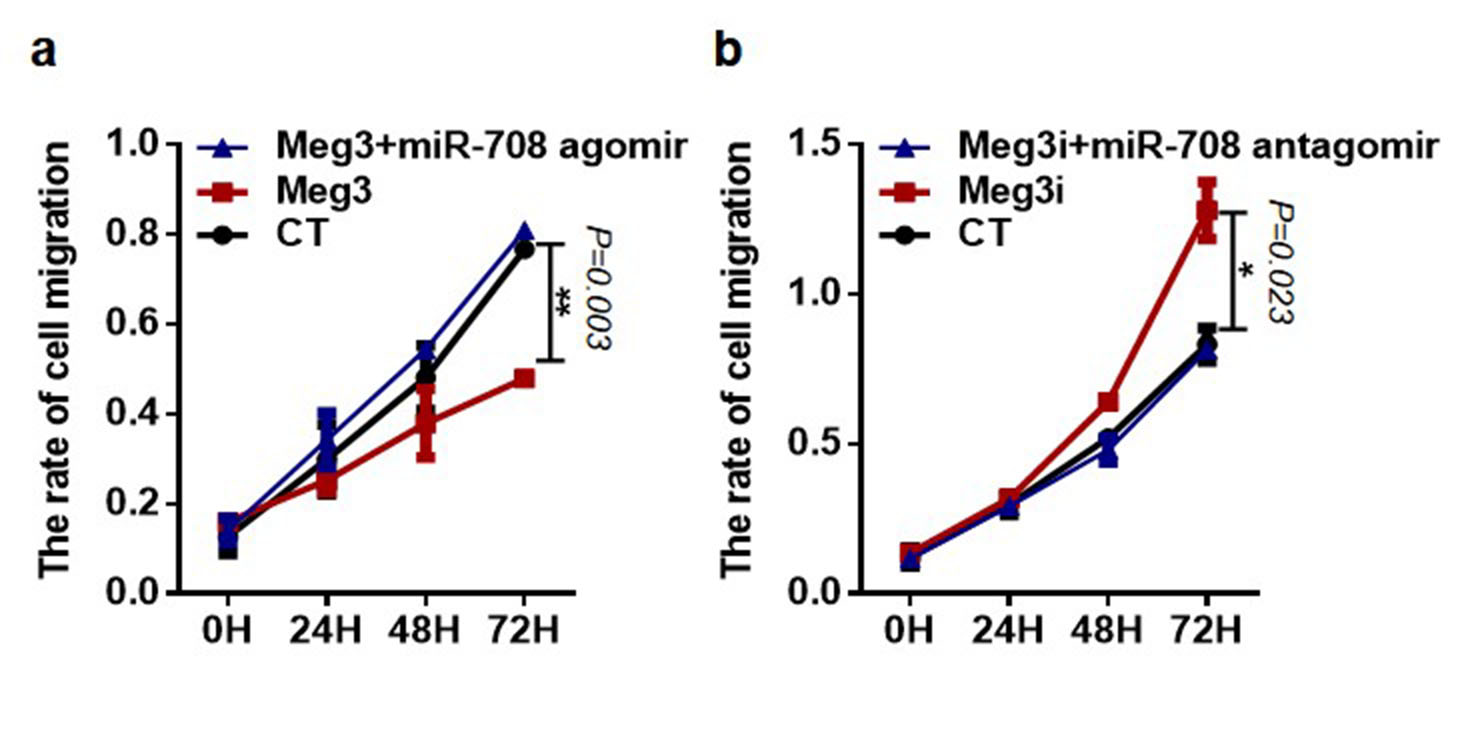

Supplement: Supplementary file 5 — Figure s4 [file 41419_2021_4470_MOESM5_ESM.tif]

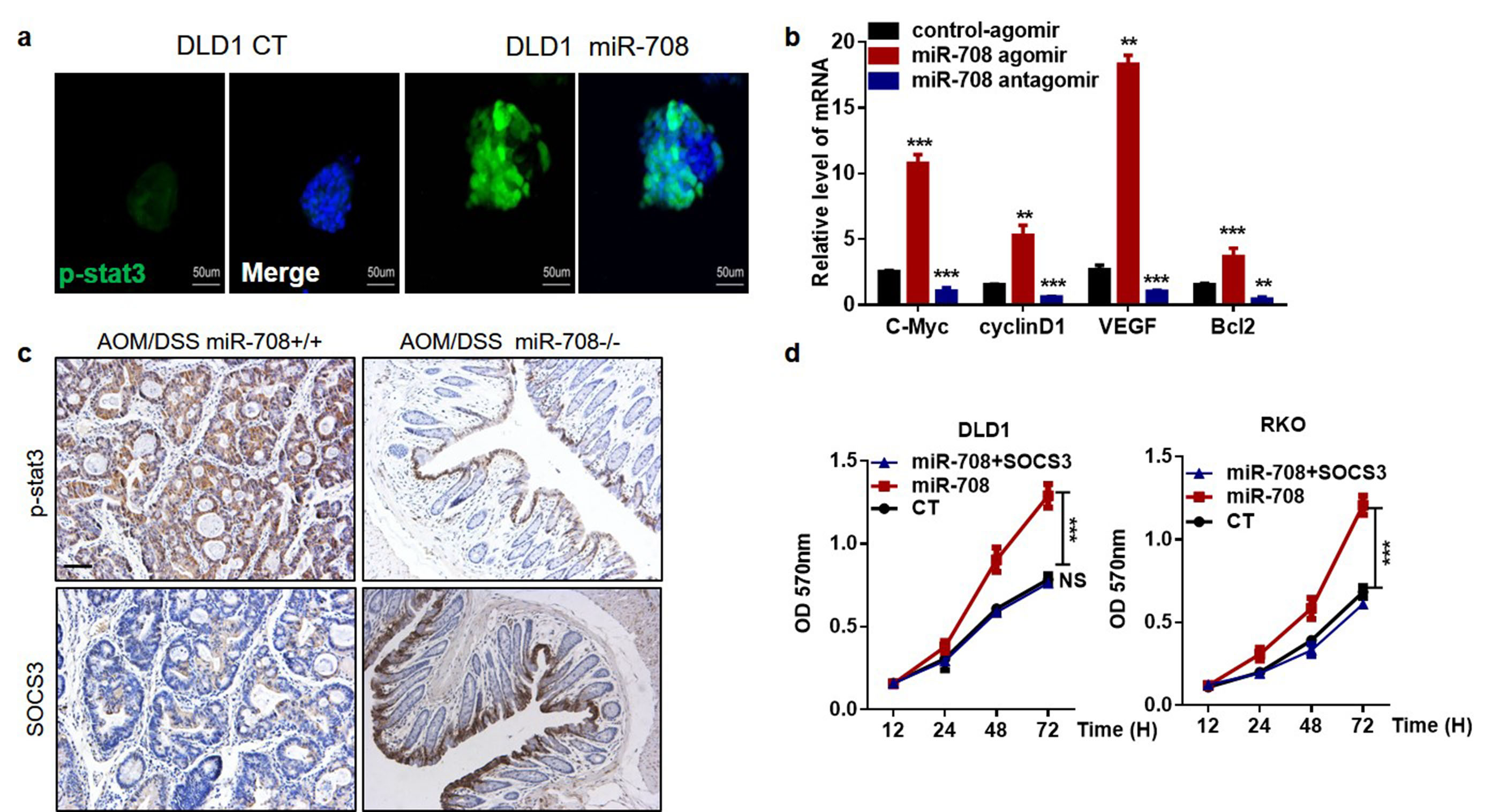

Supplement: Supplementary file 6 — Figure s5 [file 41419_2021_4470_MOESM6_ESM.tif]

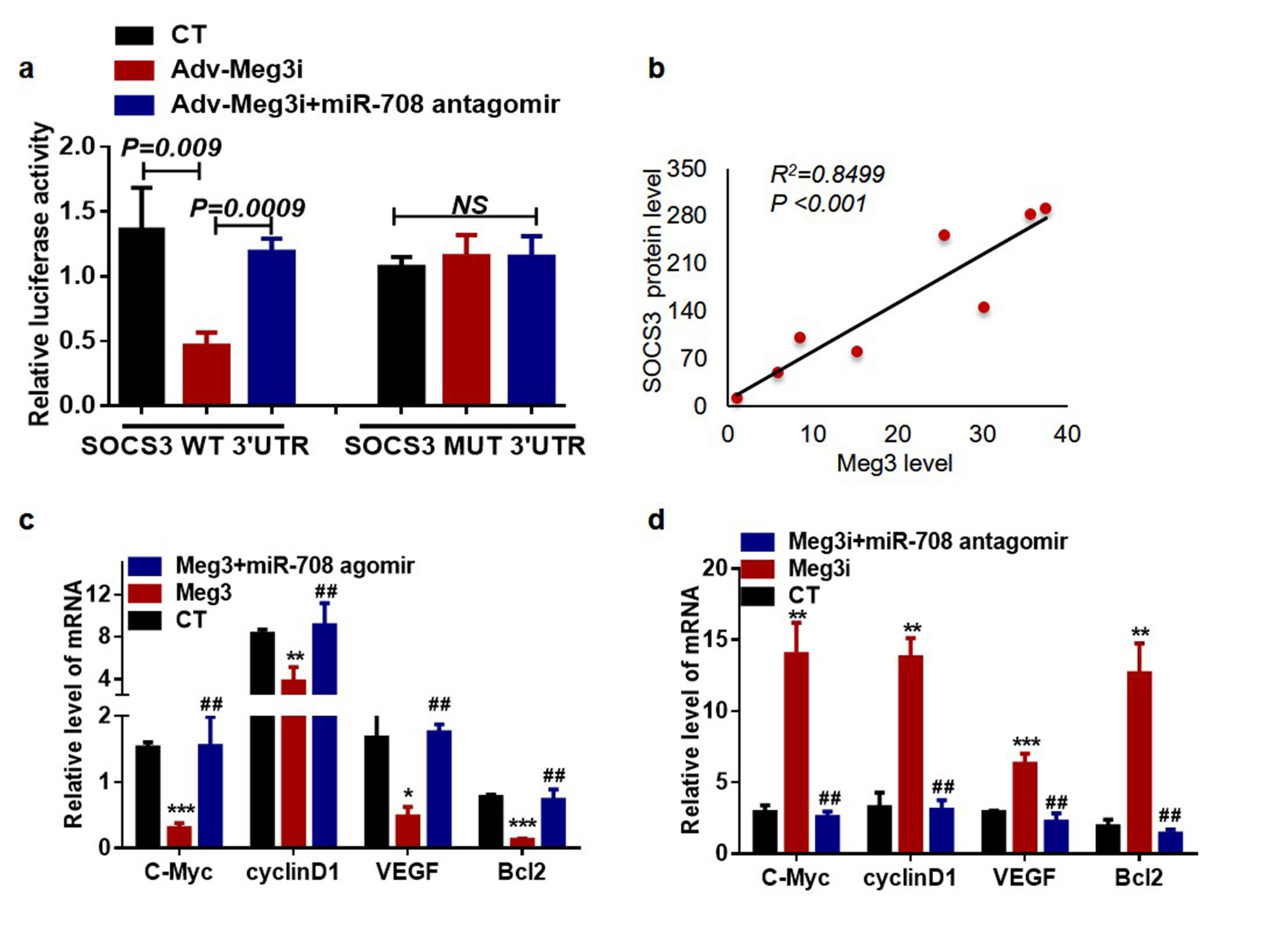

Supplement: Supplementary file 7 — Figure s6 [file 41419_2021_4470_MOESM7_ESM.tif]
